# Non-fracture acute compartment syndrome in the upper extremity of a 14-year-old boy: A case report and review of the literature

**DOI:** 10.1016/j.ijscr.2024.109983

**Published:** 2024-07-02

**Authors:** Marina Niklaus, Konrad Reinshagen, Kristofer Wintges

**Affiliations:** Department of Pediatric Surgery, University Medical Center Hamburg-Eppendorf, Martinistraße 52, 20251 Hamburg, Germany

**Keywords:** Paediatric, Emergency, Compartment syndrome, No fracture, Volkmann contracture, Case report

## Abstract

**Introduction:**

Acute compartment syndrome (ACS) is an orthopaedic emergency affecting all age groups, yet diagnosis proves particularly difficult within the paediatric population and especially in the absence of fractures.

**Case presentation:**

In this case report, we detail a rare instance of a non-fracture acute compartment syndrome (NFACS) in a 14-year-old boy, initially missed due to lack of suspicion. Symptoms included swelling, severe pain, and initial paresthesia in the hand. Despite prompt forearm fasciotomy, severe post-traumatic Volkmann contracture ensued, resulting in limited upper extremity function despite multiple corrective surgeries.

**Clinical discussion:**

Acute compart syndromes, occurring without fractures, often faces delayed diagnosis, particularly in paediatrics population. Clinical examination remains the diagnostic gold standard, with analgesia refractory pain warranting suspicion. Additional diagnostic criteria like ultrasound, MRI or CK blood values can be evaluated with reservation, especially in the paediatric population.

**Conclusion:**

This case highlights the importance of increased vigilance in diagnostics for NFACS especially in children, in order to not overlook NFACS, due to the wide variability in the aetiology and clinical appearance. We emphasize the relevance of clinical diagnostics and point out an increased awareness of NFACS in analgesic refractory pain.

## Highlights

• ACS of the extremities usually occurs in association with fractures.
• The absence of fractures can lead to misdiagnosis and thus delayed treatment.
• The gold standard is clinical examination; imaging and blood test can provide support.
• In cases of compartment syndrome, close monitoring is essential despite no fractures.
• Delayed surgical fasciotomy can lead to permanent nerve and muscle damage.

## 1 Introduction

First described by Volkmann in 1881, acute compartment syndrome is still considered to be a limb- or even life-threatening condition if not treated in a timely manner (1,2). ACS is caused by elevated pressure within a closed fascial space, leading to irreversible muscle necrosis and nerve injury due to reduced perfusion (3). Since muscle necrosis can occur after 4 h of ischemia and become permanent after eight, early diagnosis and emergency fasciotomy are essential to prevent permanent disability (4). In children, delayed diagnosis and treatment of ACS are common because its presentation can be atypical (5). Moreover, the high association of ACS with injuries and fractures can lead to decreased suspicion and thus to delayed proper diagnosis when these findings are missing at time of presentation (6). In the literature, only isolated case reports and reviews regarding NFACS in children are found, which overall emphasize the different etiologies and delayed treatment. It becomes evident that clinical examination is crucial, with analgesia resistant pain being a notable feature (5,7). In the study by Livingston et al., lasting neurological and functional deficits were described in over one-third of the patients (5). This underscores the importance of critical and prompt diagnosis in children.

We report on a 14-year-old male patient with a NFACS in the upper extremity due to delayed diagnosis because of missing suspicion, resulting in a severe post-traumatic Volkmann contracture. The work has been reported in line with the SCARE criteria (8).

## 2 Case presentation

**Fig. 1:**
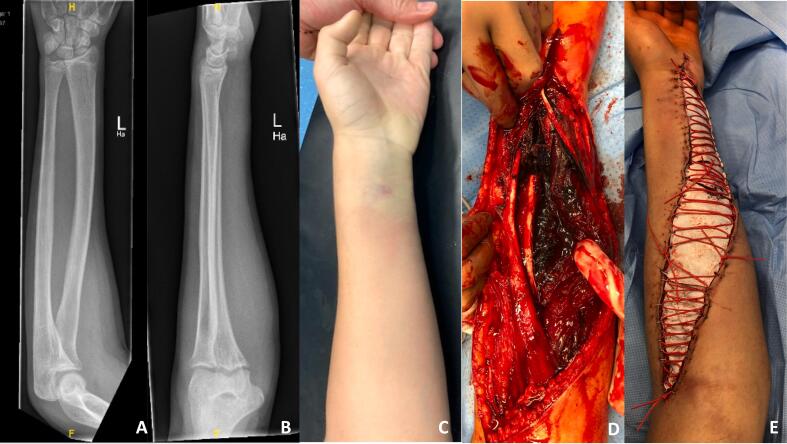
Conventional X-ray in two planes of the injured forearm of the 14-year-old boy showed no sign of fracture (**A+B**). Upon presentation to our emergency room, the left forearm and hand displayed pronounced circulatory disturbance, manifested by a pale hand (**C**). Intraoperatively, a hematoma was identified within the deep flexor tendon compartment following decompression fasciotomy (**D**). Flexor sided wound defect with “Roman sandal wound closure” with EpiGard® after decompression fasciotomy (**E**).

A 14-year-old male patient with no known pre-existing conditions presented at our emergency room with clinical signs of compartment syndrome in his non-dominant left forearm and hand after a fall during a volleyball game 18 h earlier. Informed consent of the parents and the approval of the ethics committee were obtained. Primary presentation occurred after 2 h after accident at an external hospital, with clinical symptoms of the patient's forearm including swelling, severe pain as well as beginning paraesthesia in fingers and hand. Fracture of the affected limb was excluded using conventional x-ray (Fig. 1**A+B**). He was discharged home without further diagnostics, such as an ultrasound or blood test, with persistent pain and a misdiagnosis of a bruised arm. At home, despite continued analgesic therapy, the boy's pain and paraesthesia worsened considerably overnight, and he was already struggling with nausea. This prompted his parents to seek a second opinion in our emergency department. Upon arrival at our emergency room 15 h after the initial presentation, the boy's left arm already showed a flexion contracture, hypaesthesia, and pronounced circulatory disturbance, evident by a pale hand (Fig. 1**C**). The clinical examination, together with significantly 100-fold elevated CK values (14,350 units/l) and analgesia resistant pain, immediately led to the diagnosis of acute compartment syndrome without fracture.

**Fig. 2:**
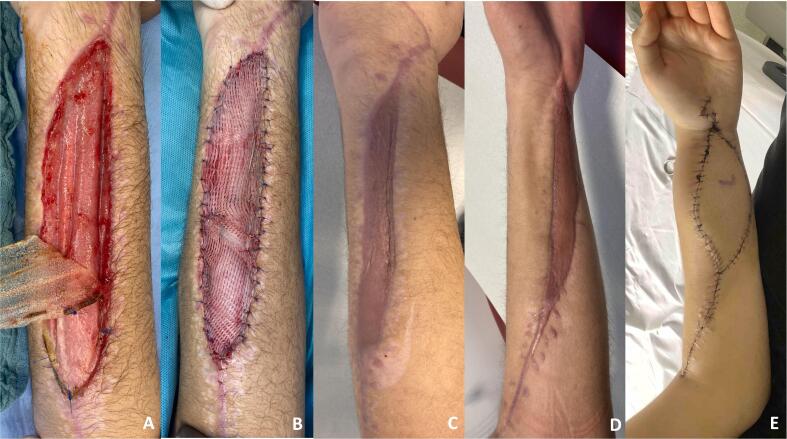
Removing the protective film of the NovoSorb® BTM (Biodegradable Temporising Matrix) after adequate healing (**A**) and covered extensor sided wound defect with split-thickness skin graft from the head (**B**). Healed split skin on the flexor and extensor side with good cosmetic results (**C+D**). The forearm was reconstructed using a free ALT flap (**E**).

An emergency fasciotomy of the forearm was performed under general anesthesia. During the procedure, the suspected cause – a hematoma within the deep flexor tendon compartment – was removed. (Fig. 1**D**). The resulting large wound defects were reduced using “Roman sandal wound closure” (Fig. 1**E**) and negative pressure wound therapy to promote healing and wound drainage was applied. Additionally, a specialized wound dressing called NovoSorb® BTM, along with split-thickness skin grafts harvested from the head, were used to cover the defects (Fig. 2**A+B**).

**Fig. 3:**
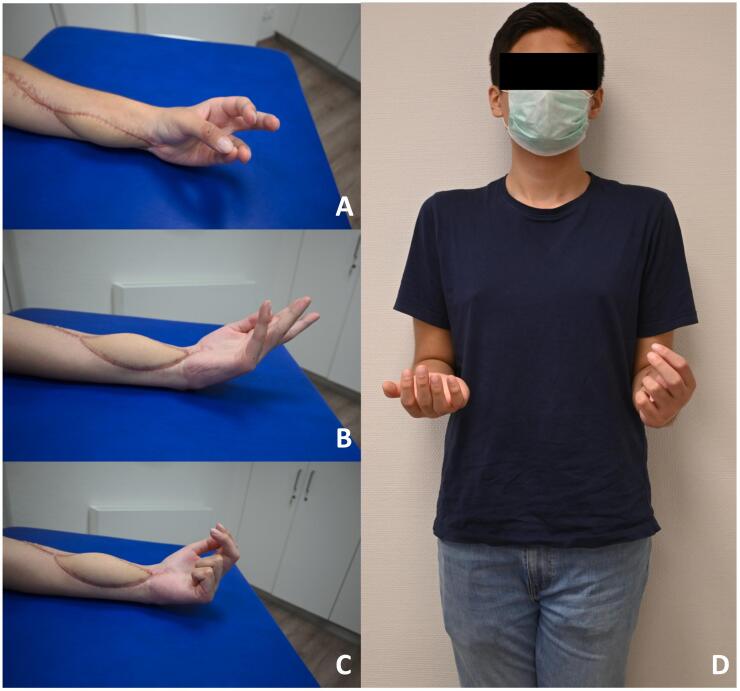
The physical examination one year following the last surgery reveals almost normal thumb opposition (**A**). However, limited mobility persist in the wrist (**B**), finger flexion (**C**) as well as forearm rotation (**D**).

At six months follow-up, good cosmetic results were observed (Fig. 2**C+D**). However, despite intensive physiotherapy and occupational therapy, the patient developed severe Volkmann's contracture with restricted wrist and forearm mobility with a DASH score of 100/100 and persistent pain (VAS 2–4.) To improve mobility, the patient was referred to a center for further treatment of Volkmann's contracture. Surgery included a necrosectomy of the flexor tendons, tendon transfers and neurolysis with an ALT flap for coverage as well as a proximal row carpectomy (Fig. 2**E**). Nearly two years after the accident, while the hand still has functional limitations, its usability in daily activities has been steadily improved. Thumb opposition has been regained (Fig. 3**A**). Wrist mobility has improved to 20 degrees of extension and 70 degrees of flexion, along with 90 degrees of pronation and 45 degrees of supination (Fig. 3 **B+D**). Finger-to-palm distance (3–3.5-0-0) and finger strength have demonstrably improved (Fig. 3**C**). The DASH score was still 79/100 but he no longer has any pain (VAS 0). Sensitivity has fully normalized. The severity of the injury and the significant limitations the patient experienced ultimately resulted in the development of a stress diosorder characterized by social withdrawal.

## 3 Discussion

Similar to adults, ACS of the extremity in the paediatric population is mainly caused by fractures (5). Therefore, especially in non-fracture injuries the attending physician might not consider ACS as possible diagnosis, leading to a delay in identification and subsequent interventions (5). Consequently, the patient's outcome might be aggravated due to the lack of early treatment resulting in an increased risk of complications, such as myonecrosis, neurological deficits, contractures, myoglobinaemia with acute renal failure or even amputation (2,4,6,9). Particularly, male adolescents are exposed to an increased risk of developing NFACS due to less compliant fascial envelopes compared to female adolescents (10).

In several studies, a delay from injury to surgical intervention comparing patients with fracture-associated compartment and NFACS has been confirmed (1,5). Overall, patients with NFACS showed a lower rate of full recovery, especially compared to cases that received treatment in <24 h (1,5,11). To avoid any of the devastating consequences, the aetiologies of ACS, particularly in the absence of fractures, should be considered. Especially, trauma-associated, iatrogenic, infectious and exercise-related events have been shown to have high relevance in the development of NFACS (1,5,6).

The clinical presentation of ACS may be variable but essential for timely diagnosis. The characteristics of the clinical symptoms are often described using the 5P's (pain, pallor, paraesthesia, paralysis, and pulselessness) with pain as the hallmark symptom regardless of the aetiology of ACS (5,12). Nevertheless, these clinical signs can appear very late, are often not fully developed and are rather unspecific (2). Noonan et al. therefore described the 3As (increasing anxiety, agitation and increasing analgesia requirement) for recognizing compartment syndrome in children (13). In fact, specifically the need for analgesia or refractory pain to analgesia appears to have a high sensitivity for NFACS (1,2). While these criteria are valuable for diagnosing ACS, accurately assessing in very young patients can be challenging due to communication difficulties and limited cooperation during physical examinations while being in pain. In addition, complimentary assessments measuring compartment firmness using digital palpation alone have shown a low sensitivity of only 24–49 % and low specificity of 55–79 % (14,15).

To ensure timely diagnosis in patients with limited communication or unclear symptoms, intracompartimental pressure measurement (ICP) is recommended (1,16,17) Most of the recommendations for ACS diagnostics, however, relate to adults and therefore cannot be applied to children (17). Due to the lack of standards in children, the use of ICP measurements is controversial, particularly regarding the pressure threshold for mandatory surgery (1,17). Furthermore, compartment measurements cannot be carried out properly in conscious children and often requires sedation or anesthesia. Therefore, the use of ICP measurement may only aid in a selected group of paediatric patients where suspicion for ACS is high. Additional imaging techniques can aid in equivocal cases. MRI and ultrasound may reveal muscle edema and swelling and disruption of the normal fascial architecture, potentially indicating compartment syndrome. These techniques can also be used to detect hematomas in the affected compartments, which can be the cause of the increase in pressure in the compartments as shown in our case report. However, ultrasound and MRI cannot definitively quantify compartment syndrome based on morphologic changes, and there is no confirmed correlation between these morphologic changes and ICP in vivo (18,19). In addition, elevated creatine kinase (CK) levels in the blood (>3000 units/l) can support the diagnosis of ACS, as Weingart et al. were able to show in a study in adults (20). Nevertheless, in children the gold standard for the diagnosis of ACS is and remains the clinical examination.

## 4 Conclusion

ACS is particularly difficult to diagnose in children. Although it is most commonly associated with fractures, other aetiologies must also be considered as current literature shows a wide variety of reasons for the development of NFACS. Furthermore, symptoms are often unspecific and difficult to evaluate in the paediatric population. However, especially analgesia refractory pain should be considered as an early indicator of NFACS. In the case of appropriate clinical symptoms, children must be closely monitored and, if suspected, urgent fasciotomy conducted.

## Abbreviations

ACS: Acute compartment syndrome
ALT flap: anteriorlateral thigh flap
APL: Abductor pollicis longus muscle
BTM: Biodegradable Temporising Matrix
CK: creatinine kinase
ECU: extensor carpi ulnaris muscle
EPB: extensor pollicis brevis muscle
EPL: extensor pollicis longus muscle
NFACS: Non-fracture acute compartment syndrome
ICP: intracompartmental pressure

## Financial disclosure

This study has not received funding from any grant giving or other bodies.

## Ethical approval

The approval of the Ethics Committee Hamburg Germany was requested and was not required due to the nature of the article (case report).

## Funding

As this manuscript was a case report with no new medical device nor surgical techniques, not prior registration is required.

There has not been any funding.

## Author contribution

Marina Niklaus: Data collection; data analysis; writing review & editing.

Kristofer Wintges: study concept, visualization and supervision.

Konrad Reinshagen: project administration and data validation.

## Guarantor

Marina Niklaus.

Kristofer Wintges.

Konrad Reinshagen.

## Research registration number

None.

## Declaration of competing interest

All authors declare that they have no conflict of interest.
